# Supplementary material for: Mental health and illness of medical students and newly graduated doctors during the pandemic of SARS-Cov-2/COVID-19
Source: PLoS One. 2021 May 18;16(5):e0251525. doi: 10.1371/journal.pone.0251525 (PMC8130957; doi:10.1371/journal.pone.0251525)
Supplement: S1 File — (DOCX) [file pone.0251525.s001.docx]

S1 File

QUESTIONNAIRE APPLIED AT THE MOMENT ONE (M1):

DADOS SOCIODEMOGRÁFICOS / SOCIODEMOGRAPHIC DATA

Qual o estágio da sua formação universitária? 9º semestre em curso; 10º semestre em curso; 11º semestre em curso; 12º semestre em curso; concluí o curso de medicina em 2018 e faço residência médica; concluí o curso de medicina em 2018 e não faço residência médica; concluí o curso de medicina em 2019 e faço residência médica; concluí o curso de medicina em 2019 e não faço residência médica; concluí o curso de medicina em 2020 e faço residência médica; concluí o curso de medicina em 2020 e não faço residência médica; concluí o curso antes de 2018 / What is the stage of your university education? 9th semester in course; 10th semester in course; 11th semester in course; 12th semester in course; I completed the medical course in 2018 and I do medical residency; I completed the medical course in 2018 and I do not do medical residency; I completed the medical course in 2019 and I do medical residency; I completed the medical course in 2019 and I do not do medical residency; I completed a medical course in 2020 and I do medical residency; I completed the medical course in 2020 and I do not do medical residency; completed the medical course before 2018.

Qual o seu sexo? / What is your gender?

Qual a sua idade? / How old are you?

Onde você reside no momento? / Where do you currently reside?

Onde a sua família reside? / Where does your family reside?

Qual o seu estado civil? / What is your marital status?

Com quem você reside? / Who do you live with?

Você tem animal de estimação? / Do you have a pet?

Você tem filho(a)? / Do you have a child?

Considerando o período a partir de 01/03/2020, com que frequência você pratica atividade física? / Considering the period from 03/01/2020, how often do you practice physical activity?

Qual a universidade que você cursa ou cursou? / Which university do you attend or attended?

INFORMAÇÕES SOBRE A COVID-19 / COVID-19 INFORMATION

A partir de 01/03/2020 você teve: diagnóstico de COVID-19 confirmada laboratorialmente; sintomas gripais; nenhum sintoma. / From 03/01/2020, you had: laboratory confirmed COVID-19 diagnosis; flu-like symptoms; no symptoms.

Você se vacinou contra a gripe (H1N1) esse ano? / Have you been vaccinated against influenza (H1N1) this year?

Você é portador (a) de alguma condição que lhe coloque dentro do grupo de risco (gestação, puerpério, doença cardíaca, hipertensão, diabetes, obesidade, asma / DPOC, doença autoimune, doença oncohematológica)? / Do you have any condition of risk group (pregnancy, puerperium, heart disease, hypertension, diabetes, obesity, asthma / COPD, autoimmune disease, oncohematological disease)?

Você considera ter informação suficiente sobre a doença COVID-19? / Do you think you have enough information about COVID-19?

Qual (is) a(s) sua(s) principal(is) fonte(s) de informação? / What are your main source (s) of information?

Com que frequência você busca informações em fontes científicas sobre a COVID-19? / How often do you seek information from scientific sources about COVID-19?

Com base nas informações que você tem até esse momento, o que você pensa a respeito da utilização de medicamentos (antimaláricos, antibióticos e imunobiológicos) no tratamento da doença? / Based on the information you have so far, what do you think about the use of medications (antimalarials, antibiotics and immunobiologicals) in the treatment of the disease?

Você sente que está sendo exposto (a) a um excesso de informações sobre a COVID-19? / Do you feel you are being exposed to an excess of information about COVID-19?

A partir do dia 01/03/2020 marque o(s) sintoma(s) que você sentiu: Dificuldade para iniciar ou manter o sono; redução do apetite; aumento do apetite; irritabilidade; pensamentos recorrentes acerca dos riscos da COVID-19; palpitação; sudorese; sensação de falta de ar; redução da energia; disfunção sexual (perda da libido, disfunção erétil); dores recorrentes (dor de cabeça, dor muscular, dor articular); desânimo ao ir trabalhar; sensação de medo ao ir trabalhar; outro sintoma (especificar); nenhum dos sintomas / From 03/01/2020, mark the symptom (s) you felt: Difficulty to start or maintain sleep; reduced appetite; increased appetite; irritability; recurring thoughts about the risks of COVID-19; palpitation; sweating; feeling of shortness of breath; energy reduction; sexual dysfunction (loss of libido, erectile dysfunction); recurrent pain (headache, muscle pain, joint pain); discouragement when going to work; feeling of fear when going to work; other symptom (specify); none of the symptoms

Você sente que a quantidade de informações disponíveis está lhe deixando mais ansioso (a)? / Do you feel that the amount of information available is making you more anxious?

Você sente que tem o apoio emocional da Universidade / local de trabalho nesse momento de pandemia? / Do you feel that you have the emotional support of the University / workplace in this pandemic moment?

Você sente que tem o apoio emocional dos colegas (faculdade ou trabalho) nesse momento de pandemia? / Do you feel that you have emotional support from colleagues (college or work) in this pandemic moment?

Com que frequência você utiliza alguma ferramenta (videochamada, telefonema, e-mail) para manter o contato social com família e amigos? / How often do you use a tool (video call, phone call, e-mail) to maintain social contact with family and friends?

Você sente medo de contrair a COVID-19? / Are you afraid of contracting COVID-19?

Você tem medo de morrer de COVID-19? / Are you afraid of dying from COVID-19?

Você sente medo de transmitir a COVID-19? / Are you afraid to transmit COVID-19?

Você tem medo que a sua situação financeira piore por causa da pandemia? / Are you afraid that your financial situation will worsen because of the pandemic?

Qual(is) medida(s) você considera mais eficaz(es) para controlar a pandemia de COVID-19? / Which measure (s) do you consider most effective to control the COVID-19 pandemic?

Você tem usado suas mídias sociais para compartilhar informações sobre COVID-19? / Have you been using your social media to share information about COVID-19?

Qual(is) mudança(s) de convívio social tomada(s) devido à pandemia atual mais impactaram no seu dia-a-dia? / Which change (s) in social life taken (s) due to the current pandemic most impacted your daily life?

HISTÓRICO DE SAÚDE MENTAL / MENTAL HEALTH HISTORY

Com que frequência que você ingere bebida alcóolica? / How often do you drink alcohol?

Com que frequência você bebe até ficar bêbado(a)? / How often do you drink until you are drunk?

Com que frequência você fuma cigarro (tabaco)? / How often do you smoke cigarettes?

Com que frequência você faz uso de drogas ilícitas? / How often do you use illicit drugs?

Se sim, qual droga ilícita você usa? / If so, what illicit drug do you use?

Você tem ou já teve algum diagnóstico de transtorno psiquiátrico? / Do you have or have you ever had a diagnosis of psychiatric disorder?

Você faz ou já fez uso de medicação psicotrópica? / Do you use or have you used psychotropic medication?

COMENTÁRIOS FINAIS / FINAL COMMENTS

Faça um comentário se desejar: / Make a comment if you wish:

QUESTIONNAIRE APPLIED AT THE MOMENT TWO (M2):

Você respondeu a pesquisa sobre saúde mental de internos de medicina e médicos recém-formados em tempos de COVID-19 (iniciada em abril/2020)? / Did you answer the survey on mental health of medical interns and newly graduated doctors in COVID-19 times (started in April / 2020)?

DADOS SOCIODEMOGRÁFICOS / SOCIODEMOGRAPHIC DATA

Qual o seu sexo? / What is your gender?

Qual a sua idade? / How old are you?

Qual o seu estado civil? / What is your marital status?

Com quem você reside? / Who do you live with?

Considerando o período a partir de 01/03/2020, com que frequência você pratica atividade física? / Considering the period from 03/01/2020, how often do you practice physical activity?

Qual a universidade que você cursa ou cursou? / Which university do you attend or attended?

Qual o estágio da formação universitária você estava em abril/2020? 9º semestre em curso; 10º semestre em curso; 11º semestre em curso; 12º semestre em curso; concluí o curso de medicina em 2018 e faço residência médica; concluí o curso de medicina em 2018 e não faço residência médica; concluí o curso de medicina em 2019 e faço residência médica; concluí o curso de medicina em 2019 e não faço residência médica; concluí o curso de medicina em 2020 e faço residência médica; concluí o curso de medicina em 2020 e não faço residência médica; concluí o curso antes de 2018 / What stage of university training were you in April / 2020? 9th semester in course; 10th semester in course; 11th semester in course; 12th semester in course; I completed the medical course in 2018 and I do medical residency; I completed the medical course in 2018 and I do not do medical residency; I completed the medical course in 2019 and I do medical residency; I completed the medical course in 2019 and I do not do medical residency; I completed a medical course in 2020 and I do medical residency; I completed the medical course in 2020 and I do not do medical residency; completed the medical course before 2018.

Qual o estágio atual (setembro/2020) da sua formação universitária? 9º semestre em curso; 10º semestre em curso; 11º semestre em curso; 12º semestre em curso; concluí o curso de medicina em 2018 e faço residência médica; concluí o curso de medicina em 2018 e não faço residência médica; concluí o curso de medicina em 2019 e faço residência médica; concluí o curso de medicina em 2019 e não faço residência médica; concluí o curso de medicina em 2020 e faço residência médica; concluí o curso de medicina em 2020 e não faço residência médica; concluí o curso antes de 2018 / What is the current stage (September / 2020) of your university education? 9th semester in course; 10th semester in course; 11th semester in course; 12th semester in course; I completed the medical course in 2018 and I do medical residency; I completed the medical course in 2018 and I do not do medical residency; I completed the medical course in 2019 and I do medical residency; I completed the medical course in 2019 and I do not do medical residency; I completed a medical course in 2020 and I do medical residency; I completed the medical course in 2020 and I do not do medical residency; completed the medical course before 2018.

INFORMAÇÕES SOBRE A COVID-19 / COVID-19 INFORMATION

Você se vacinou contra a gripe (H1N1) esse ano? / Have you been vaccinated against influenza (H1N1) this year?

A partir de 01/03/2020 você teve: Sintomas de COVID-19 com confirmação laboratorial (RT-PCR ou sorologia); Sintomas de COVID-19 sem confirmação laboratorial; Assintomático com diagnóstico laboratorial de COVID-19; Assintomático sem diagnóstico de COVID-9. / From 03/01/2020 you had: Symptoms of COVID-19 with laboratory confirmation (RT-PCR or serology); Symptoms of COVID-19 without laboratory confirmation; Asymptomatic with laboratory diagnosis of COVID-19; Asymptomatic without diagnosis of COVID-9.

Caso você tenha tido COVID-19 confirmada, como foi o seu tratamento? Tratamento domiciliar; Internação hospitalar em enfermaria / apartamento; Internação hospitalar em UTI. / If you have confirmed COVID-19, how was your treatment? Home treatment; Hospitalization in the infirmary / apartment; Hospitalization in the ICU.

Alguma pessoa próxima a você adoeceu de COVID-19? / Did anyone close to you get sick from COVID-19?

Alguma pessoa próxima a você faleceu pela COVID-19? / Did anyone close to you die from COVID-19?

Você necessitou mudar de local de residência em decorrência da pandemia? / Did you need to change your place of residence as a result of the pandemic?

A sua situação financeira piorou por causa da pandemia? / Has your financial situation worsened because of the pandemic?

Se você ainda é estudante, sente-se seguro no presente momento para retornar suas atividades acadêmicas? / If you are still a student, do you feel safe at the moment to return to your academic activities?

Se você é graduado, sente-se seguro no presente momento para retornar ao seu trabalho habitual? / If you are a graduate, do you feel safe at the moment to return to your usual job?

Você trabalhou na linha de frente da COVID-19? / Did you work on the front line of COVID-19?

Sua qualidade de vida piorou nessa pandemia? / Has your quality of life worsened in this pandemic?

Caso seja médico formado, você está satisfeito com sua atuação no cenário da COVID-19? / If you are a graduated doctor, are you satisfied with your performance in the COVID-19 scenario?

Caso seja aluno do curso de medicina, você concordou com o afastamento temporário das atividades na universidade? / If you are a medical student, did you agree with the temporary withdrawal from university activities?

Durante a pandemia a Universidade/Hospital universitário lhe ofereceu apoio emocional e/ou técnico-acadêmico? / During the pandemic, did the university / university hospital offer you emotional and / or technical-academic support?

O que você acha que a universidade/hospital universitário pode fazer para ajudar nesse momento de retomada? / What do you think the university / university hospital can do to help in this moment of return?

Qual o seu aproveitamento com aulas online? / What is your level of achievement in online classes?

Você gostaria que a universidade oferecesse mais atividades de extensão voluntária? / Would you like the university to offer more voluntary extension activities?

Se sim, sobre qual área gostaria que fosse a atividade de extensão voluntária? / If so, what area would you like the voluntary extension activity to be in?

HISTÓRICO DE SAÚDE MENTAL / MENTAL HEALTH HISTORY

Você identifica início ou incremento no consumo de álcool durante a pandemia? / Do you identify the beginning or increase in alcohol consumption during the pandemic?

Você identifica início ou incremento no consumo de cigarro (tabaco) durante a pandemia? / Do you identify the beginning or increase in cigarette consumption during the pandemic?

Você identifica início ou incremento no consumo de drogas ilícitas durante a pandemia? / Do you identify the beginning or increase in the consumption of illicit drugs during the pandemic?

Se faz uso de droga ilícita, qual (ou quais) você usa? / If you use illicit drugs, which one (or which ones) do you use?

Antes de 01/03/2020 você já tinha diagnóstico de algum transtorno psiquiátrico? / Before 03/01/2020, did you already have a diagnosis of a psychiatric disorder?

A partir de 01/03/2020 até o momento atual você teve diagnóstico de algum transtorno psiquiátrico? From 03/01/20 to the present moment, have you been diagnosed with any psychiatric disorder?

Você faz uso atualmente de medicação psicotrópica (antidepressivo, ansiolítico, estabilizador de humor, antipsicótico)? / Do you currently use psychotropic medication (antidepressant, anxiolytic, mood stabilizer, antipsychotic)?

Se faz uso de medicação psicotrópica, qual (ou quais) você usa? / If you use psychotropic medication, which one (or which ones) do you use?

Quais os principais aprendizados que a pandemia lhe trouxe? / What are the main lessons that the pandemic has brought you?

Como você imagina que será seu trabalho/curso de medicina após a pandemia? / How do you imagine your job / medical course will be after the pandemic?

COMENTÁRIOS FINAIS / FINAL COMMENTS

Faça um comentário se desejar: / Make a comment if you wish:
