# Supplementary material for: Mental health and illness of medical students and newly graduated doctors during the pandemic of SARS-Cov-2/COVID-19
Source: PLoS One. 2021 May 18;16(5):e0251525. doi: 10.1371/journal.pone.0251525 (PMC8130957; doi:10.1371/journal.pone.0251525)
Supplement: Attachment — Submitted filename: Response to Reviewers.docx [file pone.0251525.s003.docx]

**Authors’ Response to the Review Comments**

Journal: PLOS ONE

Manuscript: PONE-D-20-40515
Title of Paper: Mental health and illness of medical students and newly graduated doctors during the pandemic of SARS-Cov-2/COVID-19

Dear Editor and Reviewers,

We appreciate the efforts in carefully reviewing this manuscript in this difficult time. The authors agreed with all comments, and we made adjustments and corrections. We are grateful for the suggestions that improved the quality of our article, and believe that the revised version can meet the jornal publication requirements.

**Editor Comments:**

**1. Please ensure that your manuscript meets PLOS ONE's style requirements, including those for file naming. The PLOS ONE style templates can be found at**

**https://journals.plos.org/plosone/s/file?id=wjVg/PLOSOne_formatting_sample_main_body.pdf and https://journals.plos.org/plosone/s/file?id=ba62/PLOSOne_formatting_sample_title_authors_affiliations.pdf**

Corrections were made in order to meet PLOS ONE's style requirements.

**2. Please include additional information regarding the survey or questionnaire used in the study and ensure that you have provided sufficient details that others could replicate the analyses. For instance, if you developed a questionnaire as part of this study and it is not under a copyright more restrictive than CC-BY, please include a copy, in both the original language and English, as Supporting Information.**

A copy of the questionnaires developed as part of this study was included as Supporting Information.

**3. Please provide a sample size and power calculation in the Methods, or discuss the reasons for not performing one before study initiation.**

It was included in methods that the sample size and the population size were the same: “The population size of the study was 1000 individuals, and all of them were invited to participate by email. It was included everyone who answered the web survey and met the selection criteria.”

**4.  We note that the grant information you provided in the ‘Funding Information’ and ‘Financial Disclosure’ sections do not match.**

**When you resubmit, please ensure that you provide the correct grant numbers for the awards you received for your study in the ‘Funding Information’ section.**

The information was rectfied. We declare that the author Rosana Cipolotti received funding from Postgraduate Support Program (PROAP) - CAPES - Ministry of Education - a total of R$ 4000,00 (four thousand reais). The funders had no role in study design, data collection and analysis, decision to publish, or preparation of the manuscript.

**5. We note that you have indicated that data from this study are available upon request. PLOS only allows data to be available upon request if there are legal or ethical restrictions on sharing data publicly. For information on unacceptable data access restrictions, please see http://journals.plos.org/plosone/s/data-availability#loc-unacceptable-data-access-restrictions.**

**In your revised cover letter, please address the following prompts:**

**a) If there are ethical or legal restrictions on sharing a de-identified data set, please explain them in detail (e.g., data contain potentially identifying or sensitive patient information) and who has imposed them (e.g., an ethics committee). Please also provide contact information for a data access committee, ethics committee, or other institutional body to which data requests may be sent.**

**b) If there are no restrictions, please upload the minimal anonymized data set necessary to replicate your study findings as either Supporting Information files or to a stable, public repository and provide us with the relevant URLs, DOIs, or accession numbers. Please see http://www.bmj.com/content/340/bmj.c181.long for guidelines on how to de-identify and prepare clinical data for publication. For a list of acceptable repositories, please see http://journals.plos.org/plosone/s/data-availability#loc-recommended-repositories.**

**We will update your Data Availability statement on your behalf to reflect the information you provide.**

We completely agree. All relevant data are within the manuscript and its [Supporting Information](https://journals.plos.org/plosone/article?id=10.1371/journal.pone.0248132#sec068) files.

**Review Comments:**

**Reviewer #1: One of the big limitations is that whether the participants at M1 and M2 were the same individuals, also not know that previous condition on the participants pre-the pandemic. Another is small sample, thus, the results may not represent the actual status. Therefore, the conclusion should be revised.**

Thank you for your important comment. It was explained in methods that in order to identify the participants who had answered both questionnaires, it was asked in M2 if the participant had answered the form in M1. Pre-pandemic psychiatric condition of the participants was also accessed, and conclusions were revised.

**Reviewer #2: The paper findings are truly interesting, but the presented work requires revision before publication. In general, the paper brings a great amount of information regarding the stated objective: assess the impact of COVID-19 pandemics on the mental health of students and recently graduated doctors. Nonetheless, the paper also lacks organization when presenting their findings and reports data that was not explained in their methods and is not clearly related to their aim, such as comparing subjects that lived with or without their families. The paper has great potential, but should be clearer and focus on its objective and findings. Although a research project may be very extensive, a paper should be able to summarize findings and report them in a comprehensive way to the scientific community. Authors should also check their English.**

**ABSTRACT:
The authors should consider including more specific information about COVID-19 impacts on mental health in their introduction.**

**INTRODUCTION:
Literature Review was well done. Objective and research justification are very clear.**

**METHODS:
The authors should better explain why they considered the study a longitudinal cohort. Most of the data used to extract their conclusions were based on single time point analysis (transversal analysis). The authors should state in the methods that the work was approved by the ethical committee. The authors should consider a better way to report the questions made in M1 and M2 and also to make it clearer which questions were asked at both time-points. It would be of great benefit to explain which variables were used to characterize the population and each one were your dependent variables to analyze mental health and, if possible, why.**

**RESULTS:
Data is only presented in tables, which makes it harder to visualize informations. The authors should consider using charts to illustrate their main findings.
The authors should avoid making data interpretation in the results.
The authors should consider making it clearer what each data reported is about: population baseline, characteristics, mental health evaluation across time, etc**

**DISCUSSION:
Although the discussion is well-argued, it lacks connection between paragraphs, and sometimes comprehension is undermined. The authors should consider rearranging the sequence or adding subtitles to this section.**

**CONCLUSION:
The authors should be more specific on their conclusion in line 340**

We fully agree with the comments. Information about COVID-19 impacts on mental health was added to the abstract. In methods, after discussion among the authors, we understood that this is a cross-sectional descriptive study. We also added the approval number on the ethics committee. Variables were described, and the whole methodology has been rewritten. A figure was added to the article to facilitate understanding of the data. Results were rewritten, discussion was rearranged to provide better comprehension, and conclusions were revised.
