# Supplementary material for: Mental health and illness of medical students and newly graduated doctors during the pandemic of SARS-Cov-2/COVID-19
Source: PLoS One. 2021 May 18;16(5):e0251525. doi: 10.1371/journal.pone.0251525 (PMC8130957; doi:10.1371/journal.pone.0251525)
Supplement: Attachment — Submitted filename: Response to Reviewers.docx [file pone.0251525.s004.docx]

**Authors’ Response to the Review Comments**

Journal: PLOS ONE

Manuscript: PONE-D-20-40515R1
Title of Paper: Mental health and illness of medical students and newly graduated doctors during the pandemic of SARS-Cov-2/COVID-19

Dear Editor and Reviewers,

We appreciate the efforts in carefully reviewing this manuscript in this difficult time. The authors agreed with all comments, and we made adjustments and corrections. We believe that the revised version meets the jornal publication requirements.

**Review Comments:**

**Reviewer #2:**

- **Authors should consider to make it clearer that they are writing specifically about Brazil from line 60 and beyond.**

We agree and have included the missing information.

- **Figure 1 is very interesting, but authors should consider using percentages (%) instead of absolute values, as the figure does not make it clear if the association is positive or negative. Characteristics that had statistically relevant association appear to have similar absolute numbers in terms of quantity. One can infer that this is because the “long readin physical distance” group has a smaller n size, and therefore a smaller absolute value distance readingent a higher incidence in this group (increased %). Authors should seek to make this interpretation clearer and readingente from text reading.**

We changed the figure as suggested.

- **Authors should include a Figure 1 legend to include "*" meaning.**

Legend was included.

- **Please, check english in line 127-128.**

Phrase has been reworded.

- **Line 272-273 statement is not clear. What is the correlation between some doctors self-prescribing and an adequate access to mental health?**

The word that should have been used was "few", not "some". We wanted to say that as only a few doctors prescribed themselves, we can infer that in the rest of the cases there was assistance from a mental health specialist, since a significant percentage of the participants was using psychotropic drugs.
